# Impact of pyrolysis temperature on phosphorus plant availability in biochar—A pot experiment using ^33^P dilution

**DOI:** 10.1002/jeq2.70075

**Published:** 2025-08-30

**Authors:** Saadatullah Malghani, Sander Bruun, Muhammad Ashfaq Wahid, Dorette Sophie Müller‐Stöver

**Affiliations:** ^1^ Department of Plant and Environmental Sciences, Faculty of Science University of Copenhagen Frederiksberg Denmark; ^2^ Department of Agronomy University of Agriculture Faisalabad Faisalabad Pakistan

## Abstract

Separation and pyrolysis of the solid fractions of biogas digestate and animal slurry offer potential solutions to environmental and logistical challenges associated with direct slurry application as fertilizer. However, thermochemical transformations during pyrolysis typically reduce P availability. This study evaluated biochars produced at 400°C, 500°C, and 600°C from the solid fractions of biogas digestate (BDF) and pig manure (PMF) for their P‐fertilization effects using a pot experiment with perennial ryegrass (*Lolium perenne* var. Soriento) and the ^33^P dilution approach. The ryegrass biomass across two harvests remained similar for all biochar treatments but was significantly lower than for the mineral fertilizer (KH_2_PO_4_) treatment. Significant differences were evident in P contribution from biochars and raw feedstocks, as well as in total P uptake rates between treatments. The readily available P contents of biochar and P‐recovery rates in plant shoots were negatively correlated with pyrolysis temperature, which was especially pronounced for digestate‐derived biochars. All materials except high‐temperature biochar (600°C) had mineral fertilizer replacement values exceeding 50%, indicating substantial P‐recycling potential. Biochars produced at 400°C and 500°C had a similar fertilizer value as their original feedstocks. Therefore, low‐temperature pyrolysis of separated solid fractions represents a promising approach that preserves the P fertilizer value while providing climate benefits through soil C sequestration and reduced energy requirements for transport.

## Core Ideas

- Pyrolysis of P‐rich manure and digestate could mitigate environmental and logistical challenges.
- P‐rich biochar retains sufficient phosphorus to support plant growth.
- Plant‐available P decreases with increasing pyrolysis temperature.
- Low‐temperature biochar has a P fertilizer value comparable to raw feedstock.

## Plain Language Summary

Instead of applying raw agricultural waste directly to fields, converting it into biochar through heating (pyrolysis) can improve waste management. To test this, we produced biochar from the solid fractions of separated pig manure and biogas digestate at 400°C, 500°C, and 600°C and used it as a phosphorus (P) source in a pot experiment. We measured P uptake by ryegrass and, using a radioisotope method, determined how much P came from soil, seeds, and fertilizers. The results showed that biochar produced at lower temperatures (400°C–500°C) provided P as effectively as raw waste. However, biochar produced at 600°C had a significantly lower fertilizer value. This suggests that pyrolysis can enhance agricultural waste recycling, but the temperature must be carefully controlled to preserve biochar's effectiveness as a fertilizer.

**Abbreviations**
ANOVA: analysis of variance
BDF: solid fractions of biogas digestate
DGT: diffusive gradients in thin films
FPR: fertilizer phosphorus recovery
HSD: honestly significant difference
Pdf: phosphorus derived from
P‐FRV: phosphorus fertilizer replacement value
PMF: solid fractions of pig manure
TP: total P
WEP: water extractable phosphorus
WHC: water holding capacity

## 1 INTRODUCTION

Phosphorus (P) is an essential macronutrient for plants, but despite the large stock of P in agricultural soils, its low solubility and limited plant availability are major constraints for crop growth and yield. To overcome this problem, mineral P application is widely practiced globally. The primary source of P fertilizers is non‐renewable phosphate rock, which is geographically concentrated in specific regions (Brownlie et al., 2023). To ensure future food security amid P scarcity requires efficient recovery and reuse of resources such as manure and sewage sludge (Gunther et al., 2018).

Animal slurry and manure‐based biogas digestate hold great promise as a sustainable P fertilizer source for agriculture (Delin, 2016). While they are already used as fertilizers, their direct application poses challenges in modern farming. In particular, the high water content creates economic and logistical challenges for the redistribution of P from high to deficit regions (Kleinman et al., 2002). Additionally, contaminants such as antibiotic residues, pathogens, and micro‐pollutants may pose a threat to the environment (Rathnayake et al., 2023). The storage and land application of the digestate also causes problems in terms of methane and nitrous oxide emissions, both of which are potent greenhouse gases.

To address environmental, economic, and health concerns, physical separation of slurry (e.g., by screw press) into an nitrogen (N)‐rich liquid and a P‐rich solid fraction has been suggested (Hjorth et al., 2010; Kratz et al., 2019). This approach reduces the organic matter in the liquid fraction, thereby lowering methane emissions during storage and minimizing ammonia volatilization during land application (Fangueiro et al., 2008; Martinez et al., 2003; Sommer & Hutchings, 2001). The solid fraction can then be pyrolyzed to produce biochar, which eliminates organic contaminants, stabilizes carbon (C) for long‐term sequestration, and reduces volume for transport.

The literature investigating the effects of pyrolysis conditions on the solid fraction of raw manure or anaerobic digestate suggests that nearly 100% of the P can be recovered in the resulting biochar (Christel et al., 2014; Liu et al., 2023). However, the inorganic and organic P present in the feedstock is thermally modified into a complex mixture of P species, which results in reduced P availability (Tesfaye et al., 2021). Few studies have investigated the P fertilizer effect of biochars derived from the solid fraction of manure or digestate in plant‐based systems. Recently, Kopp et al. (2023) and Pedersen et al. (2025) tested the P fertilizer efficiency of biogas digestate‐derived biochars (pyrolyzed at about 550°C–600°C), showing variable effects depending on feedstock, particle size, and soil type. However, a realistic assessment of the biochar P fertilizer value and support for decision‐making requires comparison with both mineral fertilizer and the amount of feedstock used to produce the biochar (Rathnayake et al., 2023).

Biochar has also been shown to affect the P dynamics in soil indirectly via alterations to pH and sorption capacity, or by affecting soil microbes such as arbuscular mycorrhizal fungi (F. Li et al., 2019). To separate these effects from the direct uptake from the biochar fertilizer, an indirect radioisotope (^33^P) labeling approach of the soil P pool can be applied (Fardeau et al., 1995).

The present study investigated the direct contribution of the solid fraction of pig manure or biogas digestate to plant P uptake, as affected by different pyrolysis temperatures. A pot experiment was conducted using biochar as a direct source of P for the growth of perennial ryegrass to assess the P fertilization potential of six biochars produced from two different feedstocks, each pyrolyzed at temperatures of 400°C, 500°C, and 600°C. We hypothesized that biochar would serve as a direct P source for plants, and that the P fertilization effect of biochar would be negatively affected by increasing pyrolysis temperature.

## 2 MATERIALS AND METHODS

### 2.1 Characteristics of soil, pig manure, biogas digestate, and their biochars

The soil used in the experiment was obtained from a long‐term Nutrient Depletion Trial located at the experimental farm of the University of Copenhagen in Taastrup, Denmark (55°40́ N, 12°17́ E). This soil, categorized as Luvisol according to the FAO classification, was a sandy loam containing 16.4% clay and 17.3% silt. The total C, N, and P contents per kilogram of dry soil were 11.5 g, 1.55 g, and 341 mg, respectively. For the pot experiment, the soil was modified by adding 25% sand to further dilute the P content.

The pig slurry and the biogas digestate slurry (pig slurry as primary) were provided by Aarhus University, Foulum, and processed using a screw press separator at Højgaards, Denmark. The resulting solid fractions of biogas digestate (BDF) and pig manure slurry (PMF) were then pyrolyzed at the Technical University of Denmark at 400°C, 500°C, and 600°C (1 h residence time, 5°C min^−1^ heating rate). The raw solid fractions are referred to as BDF and PMF, and the six resulting biochars derived from BDF and PMF are reported as BB and PB, respectively, with the suffix indicating the pyrolysis temperature (e.g., BB400, PB500).

All eight materials used in this study were characterized using different analytical techniques. Total C and N were determined by a CN analyzer (Elementar Analysensyteme GmbH), and total N and NH_4_‐N in the wet fibers were additionally determined in an external laboratory (OK Laboratorium for Jordbrug). Total P, calcium (Ca), magnesium (Mg), aluminum (Al), iron (Fe), sodium (Na), and potassium (K) were determined by inductively coupled plasma optical emission spectroscopy (Optima 5300 DV, Perkin Elmer) after microwave‐assisted single‐step digestion with HNO_3_, H_2_O_2_, and HF (modified from T. H. Hansen et al., 2013), with differences in adding HF and conditions (temperature and pressure).

To characterize the inorganic P pools in the materials, a sequential extraction (modified from Tiessen & Moir, 2007) was performed in triplicate using four extractants: deionized H_2_O, 0.5 M NaHCO_3_, 0.1 M NaOH, and 1 M HCl. The water extractable phosphorus (WEP) and bicarbonate‐extracted fractions represent the labile P pool, whereas alkali and acid‐extracted P represent less labile pools, such as P associated with Al + Fe and Ca (Tiessen & Moir, 2007). Briefly, **∼** 0.25 g of each homogenized sample was dried (65°C, 48 h), milled, and sequentially extracted with 30 mL each of deionized H_2_O, 0.5 M NaHCO_3_, 0.1 M NaOH, and 1 M HCl. For each extraction, samples were shaken (end‐over‐end, 16 h), centrifuged (5000 g, 5 min), and filtered. The ortho‐P in the supernatant was analyzed by the molybdenum blue method using a flow injection analyzer (FIA star 5000, Foss Analytical). The pellet and the filtered retained material proceeded to the next extraction. The amount of total P (TP) not recovered in the sequential extracts was classified as residual P.

**Core Ideas**

- Pyrolysis of P‐rich manure and digestate could mitigate environmental and logistical challenges.
- P‐rich biochar retains sufficient phosphorus to support plant growth.
- Plant‐available P decreases with increasing pyrolysis temperature.
- Low‐temperature biochar has a P fertilizer value comparable to raw feedstock.

### 2.2 Establishment and maintenance of the pot experiment

We employed the indirect isotope labeling approach to quantify plant P uptake from target P sources. A detailed description of the method can be found in Frossard et al. (2011). In the present study, we performed the following steps:

#### 2.2.1 Soil pre‐treatments (nutrient solutions and ^33^P labeling of available P pool in the soil)

First, for each pot, approximately 1 kg (dry weight) of 2 mm sieved and thoroughly mixed soil was placed in a double layer of ziplock plastic bags and P‐free nutrient solutions were added. These solutions were added in quantities to provide the following amounts of nutrients per kilogram of soil: 150 mg N, 100 mg K, 25 mg Mg, 115 mg sulfur (S), 30 mg Ca, 0.45 mg manganese (Mn), 0.3 mg zinc (Zn), 0.15 mg copper (Cu), 0.01 mg molybdenum (Mo), 0.22 mg boron (B), and 0.3 mg Fe. The solutions were prepared from the following salts: NH_4_NO_3_, K_2_SO_4_, CaCl_2_, KCl, MgSO_4_7(H_2_O), MnSO_4_H_2_O, ZnSO_4_7(H_2_O), CuSO_4_5(H_2_O), Na_2_MoO_4_, H_3_BO_3_, and C_10_H_12_FeN_2_NaO_8_. To ensure uniform distribution, the soil was air‐dried for 4 days after the addition of the solutions and then thoroughly mixed. After this procedure, the moisture content of the soil was adjusted to 30% of the water holding capacity (WHC) and the samples were pre‐incubated at room temperature for 1 week.

Each soil sample was then radioactively labeled by adding 5 mL of carrier‐free ^33^P‐orthophosphate solution to achieve an activity of 2.2 MBq kg^−1^ of soil. To obtain near‐equilibrium conditions for ^31^P and ^33^P, the soil samples were thoroughly mixed for 2 min in a separate container and then incubated for 1 week at room temperature. To calculate the required amount of ^33^P needed, the radioactive ^33^P solution was measured by scintillation counting using a Liquid Scintillation Analyzer (Tri‐Carb 2910 TR, PerkinElmer) with 5 mL of solution and 15 mL of scintillation liquid (Ultima Gold).

#### 2.2.2 Experimental treatments and ryegrass sowing

The pot experiment consisted of 10 treatments (four replicates each): two controls (NoP without P addition and MinP with mineral P as KH_2_PO_4_), two raw feedstocks (BDF and PMF), and six biochars (BB400, BB500, BB600, PB400, PB500, and PB600). All treatments except NoP received equal amounts of total P to ensure comparability of P sources. Raw feedstocks (70% moisture content) and biochars were sieved (<2 mm) before soil application.

After ^33^P/^31^P equilibration (1 week), all materials including MinP were applied at 50 ± 2 mg P kg^−1^ soil. Materials were thoroughly mixed into the soil and transferred to pots (12 cm diameter, 11.7 cm height, closed bottoms). The initial N applications in the BDF and PMF treatments considered their NH_4_^+^ contents (**∼**60 mg N per pot) to ensure similar nutrient levels across treatments. Due to observed N deficiency symptoms (probably caused by N‐immobilization), this amount of N was later added to compensate.

The soil moisture in the pots was adjusted to 60% WHC and 1.5 g seeds of perennial ryegrass (*Lolium perenne* var. Soriento), containing 3.74 ± 0.11 mg P, were sown evenly into each pot and covered with 30 g of soil that had been separated while filling the pots.

To estimate the seed contribution to plant uptake, a parallel experiment with perennial ryegrass was conducted using sand and different amounts of water‐soluble P labeled with ^33^P (Nanzer et al., 2014). In brief, 1 kg of sand was amended with 1.6, 3.1, 6.3, 12.5, and 25 mg^−1^ P kg^−1^ using KH_2_PO_4_ with a specific activity of 130, 65, 33, 16, and 8 KBq mg^−1^ P, respectively.

Each treatment in the sand experiment also had four replicates and fertilization with P‐free nutrient solution, and the handling of pots followed the same procedures as described for the soil‐based experiment.

#### 2.2.3 Maintenance of plants growth conditions and harvesting

All pots (soil and sand) were maintained under controlled conditions in a growth chamber with the following settings: daylight period of 16 h, temperature of 20°C/15°C (day/night), and photosynthetically active radiation of 300/0 µmol m^2^ s^−1^ (day/night). Pots were randomly placed and repositioned regularly to reduce variation induced by environmental effects. Soil moisture was maintained at 60% WHC by daily watering with deionized water.

The ryegrass shoots were cut twice 3 cm above the soil surface: at 31 days (first cut) and 63 days (second cut) after germination. After the first harvest, a nutrient solution was applied to each pot, containing (per kg of soil): 100 mg N, 100 mg K, 20 mg Mg, and 20 mg Ca (added as NH_4_NO_3_, K_2_SO_4_, MgSO_4_ and CaCl_2_).

#### 2.2.4 Plant biomass, P contents, and ^33^P activity

The dry plant biomass was determined after drying the harvested plant samples in an oven at 60°C for 48 h. To determine the P concentration in plant samples, the muffle furnace method was used. Briefly, 500 mg of thoroughly mixed, milled plant sample was placed in acid‐washed porcelain crucibles, transferred to a muffle furnace, and kept at 550°C for 1 h. After briefly cooling the furnace, each sample was transferred from the crucible to Falcon tubes using 50 mL of freshly prepared 0.5 M H_2_SO_4_. The crucible was washed several times to ensure that the whole sample was transferred to the Falcon tube. The samples were then thoroughly mixed using an end‐over‐end shaker for 16 h before filtration and analysis.

Aliquots of each sample were used to measure P content and ^33^P activity. The P content was measured using a continuous flow injection analyzer (FIA star 5000, Foss Analytical), while the ^33^P beta‐emission of the plant extract was measured by scintillation counting (Liquid Scintillation Analyzer Tri‐Carb 2910 TR, PerkinElmer) with 5 mL of extract and 15 mL of scintillation liquid (Ultima Gold).

#### 2.2.5 Calculations for partitioning of P taken up by plants and estimating the P‐replacement values of the soil amendments

The values of P contents in shoot biomass and the total biomass were used to calculate the total P uptake (mg) per pot.

$$P_{\mathrm{uptake}}=P_{\mathrm{biomass}}\times \mathrm{Biomas}s_{\mathrm{plant}}$$

where P_biomass_ is the P concentration of P in the biomass (mg P g^−1^) and Biomass_plant_ is the total dry aboveground biomass (g).

The P uptake (mg P kg^−1^ soil) represents the cumulative contribution of P derived from (Pdf) different P sources. In the soil experiment, there were three potential sources:

$$P_{\mathrm{uptake}}=\mathrm{Pd}f_{\mathrm{soil}}+\mathrm{Pd}f_{\mathrm{fertilizer}}+\mathrm{Pd}f_{\mathrm{seed}}$$

where P_uptake_ (mg P kg^−1^) is the total P taken up in aboveground plant shoots, and Pdf_soil_, Pdf_fertilizer_, and Pdf_seed_ represent the fraction of P_uptake_ derived from soil, fertilizer (additional P source), and seeds, respectively.

In general, the contribution of Pdf_seed_ is estimated using a function derived from the correlation between P_uptake_ and Pdf_seed_ in each cut of the sand‐based parallel experiment (Nanzer et al., 2014). However, in the present study, this approach was applied only to the first cut (Pdf_seed _= 1.22+0.47 × P_uptake_ *_,_*), while Pdf_seed_ was assumed to be 0 for the second cut (Figure S1). The primary reason for limiting the function to the first cut was that the calculated Pdf_seed_ values achieved 100% contribution of P added via seeds. Consequently, applying the function to the second cut would have resulted in Pdf_seed_ values exceeding 100%, leading to an overestimation of Pdf_seed_.

After correcting the P uptake values for the seed contribution (Pdf_seed_), P derived from fertilizer (Pdf_fertilizer_) was calculated as:

$$\mathrm{Pd}f_{\mathrm{fertilizer}}=\left[\left(1-\left\{\frac{{\mathrm{SA}}_{\mathrm{treatment}}}{{\mathrm{SA}}_{\mathrm{NoP}}}\right\}\right)\times 100\%\right]$$

where SA is the specific activity (^31^P/^33^P, kBq mg^−1^ P) measured in shoots of ryegrass (treatment vs. non‐fertilized control NoP). All measured ^33^P values were decay‐corrected to the labeling day using the formula for radioactive decay (half‐life 25.34 days).

The fertilizer P recovery (FPR, %) for each soil amended P source was calculated by comparing the Pdf_fertilizer_ to the amount of P applied.

$$\mathrm{Fertizer}\;P\;\mathrm{Recovery}\;\;\left(\mathrm{FPR}\right)=\frac{\mathrm{Pd}f_{\mathrm{fertilizer}}}{P_{\mathrm{applied}}}\times 100\%$$

The P fertilizer replacement value (P‐FRV) (%) of each soil amendment applied as a P source was calculated by comparing the Pdf_fertilizer_ per unit P applied in the treatment with Pdf_fertilizer_ per unit P applied as mineral fertilizer (KH_2_PO_4_, MinP treatment).

$$\begin{array}{ccc} & & P\;\mathrm{fertilizer}\;\mathrm{replacement}\;\mathrm{value}\;\;\left(P-\mathrm{FRV}\right)\\ & & \;=\;\frac{{\mathrm{Pdf}}_{\mathrm{fertilizer}}\;\left(\mathrm{treatment}\right)}{{\mathrm{Pdf}}_{\mathrm{fertilizer}}\;\left(\mathrm{MinP}\right)}\times 100\% \end{array}$$

#### 2.2.6 Statistics and graphical presentation

Statistical analyses were carried out using R version 4.2.0 (R Core Team, 2023) and the RStudio development environment version 1.4.1106 (RStudio Team, 2023). Data normality was confirmed with a Shapiro test, and if the normality test failed, the data were transformed using the natural log or square root. To assess statistical differences, a one‐way analysis of variance (ANOVA) was performed, followed by Tukey's Honestly Significant Difference (HSD) test. In addition, linear regression analysis was performed between pyrolysis temperature (400°C, 500°C, 600°C) and various measured parameters for each feedstock separately. The graphical representation of the results was achieved using the ggplot2 R‐package (Wickham, 2016).

## 3 RESULTS

### 3.1 Characteristics of the raw materials and their biochars

Pyrolysis of biogas digestate (BDF) and pig manure (PMF) solid fractions significantly increased (*p* < 0.05) concentrations of key elements (C, N, P, Ca, Mg, Fe, and Al) in biochars compared to raw feedstocks (Table 1; Table S1). Total P in biochars was two to three times higher than in the original materials.

**TABLE 1:** Elemental composition of biochars produced from biogas digestate (BDF) and pig manure slurry (PMF) fiber after physical separation at 400°C, 500°C, and 600°C pyrolysis temperatures.

Material	Total C % (DM)	Total N % (DM)	Ash % (DM)	TP % (DM)	Ca mg g^−1^ (DM)	Mg mg g^−1^ (DM)	Fe mg g^−1^ (DM)	Al mg g^−1^ (DM)
BDF	47.6 ± 0.4f	1.04 ± 0.01e	5.6 ± 0.3g	0.39 ± 0.01d	13.1 ± 0.6c	3.7 ± 0.1c	0.45 ± 0.03b	1.1 ± 0.1c
BB400	57.6 ± 0.1e	2.05 ± 0.00a	19.5 ± 0.8c	0.79 ± 0.02bc	29.7 ± 0.6b	7.9 ± 0.2b	0.97 ± 0.02a	2.5 ± 0.1ab
BB500	61.0 ± 0.2d	2.00 ± 0.01a	21.3 ± 0.3b	0.95 ± 0.08ab	35.4 ± 2.8b	9.5 ± 0.8b	1.13 ± 0.13a	2.9 ± 0.3a
BB600	63.0 ± 0.6c	1.81 ± 0.02b	23.3 ± 0.2a	1.13 ± 0.10a	42.5 ± 3.6a	11.4 ± 1a	1.25 ± 0.23a	3.5 ± 0.3a
PMF	48.3 ± 0.2f	0.92 ± 0.07f	3.9 ± 0.03h	0.25 ± 0.04d	6.1 ± 1.2d	1.7 ± 0.2d	0.13 ± 0.05c	0.4 ± 0.2d
PB400	64.8 ± 0.7b	1.68 ± 0.01c	10.1 ± 0.6f	0.65 ± 0.11c	16.2 ± 2.6c	4.3 ± 0.7c	0.31 ± 0.03b	1.2 ± 0.2c
PB500	70.9 ± 0.1a	1.67 ± 0.01c	11.9 ± 0.1e	0.74 ± 0.12bc	18.7 ± 2.9c	5.0 ± 0.7c	0.34 ± 0.06b	1.4 ± 0.1c
PB600	72.0 ± 0.7a	1.50 ± 0.01d	13.3 ± 0.2d	0.80 ± 0.04bc	20.4 ± 0.1c	5.6 ± 0.1c	0.38 ± 0.01b	1.4 ± 0.02bc

*Note*: Values with different letters indicate statistical significance (Tukey HSD, *p* < 0.05, *n* = 3). Total N and Total C for the raw material were measured after oven drying that could cause loss of NH_4_^+^ and volatile C fractions. DM, dry matter.

BDF‐derived biochars had significantly higher mineral element and ash concentrations than PMF‐derived biochars (Tukey HSD, *p* < 0.05), though P concentrations were comparable. Despite similar levels of C in the feedstock, PMF‐derived biochars had higher C contents at equivalent temperatures.

**FIGURE 1:**
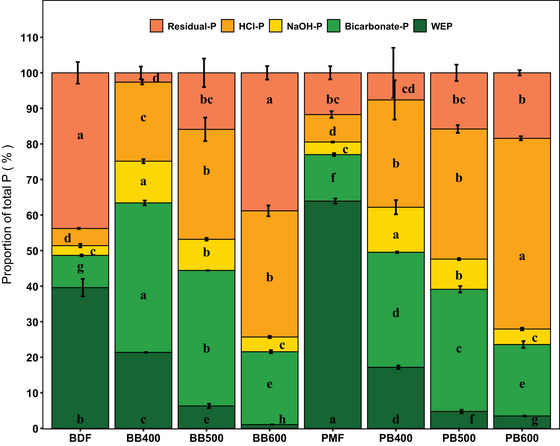
The proportions of different inorganic P fractions (sequential extractions) with respect to total P in raw fibers of biogas digestate and pig manure slurry obtained by physical separation and their biochars pyrolyzed at 400°C, 500°C, or 600°C. Water extractable phosphorus (WEP), bicarbonate‐P, NaOH‐P, HCl‐P, and residual‐P represent P fractions sequentially extracted with water, 0.5 M NaHCO3, 0.1 M NaOH, and 1 M HCl. Residual P represents P not accounted for in the sequential extraction. Different stacks in each column represent the mean values and error bars represent the standard deviation (*n* = 3). Different letters within each stack or above the error bar of each stack indicate significant statistical differences (TukeyHSD, *p*< 0.05).

Sequential P extraction recovered 53%–100% of the total P, with the lowest recovery in BDF (56.2 ± 3.0%) and the highest in BB400 (97.4 ± 1.8%; Figure 1). All P fractions (bicarbonate, NaOH‐P, and HCl‐P) were considerably higher in biochars than feedstocks, except WEP, which was lower (Tukey HSD, *p* < 0.05). Alkali‐extracted P did not differ between high‐temperature biochars (BB600 and PB600) and raw feedstocks.

The first three sequentially extracted P‐fractions, except bicarbonate‐P in PMF‐derived biochars, showed a significant decreasing trend with pyrolysis temperature, while HCl‐P had a positive trend (*p* < 0.05, Table S2). Biochars produced at 400°C had the highest proportion of P as bicarbonate‐P (32%–43%), followed by HCl‐P (26%–36%), WEP (17%–21%), and NaOH‐P (11%–14%). In contrast, biochars produced at 600°C had the largest fraction of P as HCl‐P (34%–54%), followed by bicarbonate‐P (19%–21%), NaOH‐P (4%–5%), and WEP (1%–4%).

### 3.2 Ryegrass biomass and P‐contents

**FIGURE 2:**
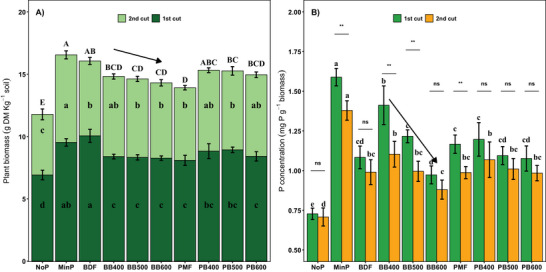
Plant biomass and P concentrations in pots amended with raw fibers from biogas digestate and pig manure or their biochars produced at three contrasting pyrolysis temperatures. (A) Dry matter content of ryegrass harvested twice. Each stack represents the mean value of each harvest, and the error bars represent the standard deviation. Different lowercase letters refer to statistically significant differences within each harvest, whereas different uppercase letters represent statistically significant differences in total biomass (Tukey HSD, p < 0.05). (B) P concentration of ryegrass biomass at each harvest. Different letters indicate statistical significance (Tukey HSD, *p* < 0.05) within each harvest, while asterisks or “ns” refer to statistically significant or non‐significant differences between the two harvests, respectively. The arrows above the columns representing biochars indicate a statistically significant linear relationship with pyrolysis temperature.

The plant biomass of the two harvests at days 31 and 63 was significantly higher in the P‐amended treatments than in the NoP treatment (ANOVA, *p* < 0.001; Figure 2A). MinP and BDF produced the highest total biomass (16.5 g kg^−1^ soil), followed by PB400 and PB500 (both ∼15.3 g kg^−1^ soil). All biochar treatments except PB400 had significantly lower biomass than MinP (Tukey HSD, *p* < 0.05), and PMF biomass was significantly lower than MinP, BDF, PB400, and PB500 (Tukey HSD, *p* < 0.05). A linear increase (*p* < 0.05) in biomass was evident with increasing P concentration in the sand experiment (Figure S2).

Phosphorus concentrations of ryegrass biomass differed significantly among treatments (ANOVA, *p* < 0.001; Figure 2B). BB400 had the highest P concentration among all biochar treatments, and BB600 had the lowest. Concentrations in treatments with BDF‐derived biochars decreased with pyrolysis temperature (*p* < 0.001), while those in treatments with PMF‐derived biochars showed no significant trend, suggesting the importance of feedstock type.

### 3.3 Contribution of different P sources to total P uptake

**FIGURE 3:**
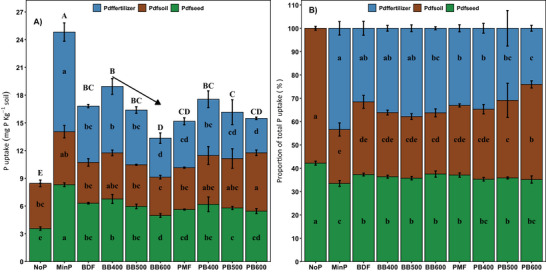
The source partitioning of the P taken up by the ryegrass during the two harvests in pots amended with different P sources (P derived from amendments, Pdf_fertilizer_, P derived from soil, Pdf_soil_, and P derived from seeds, Pdf_seed_. The proportion of Pdf_seed_ was calculated using a function derived from a separate experiment, whereas the other two fractions were calculated using the indirect ^33^P approach. (A) The amount of P (mg P kg^−1^ soil) taken up by ryegrass shoots during the two harvests and (B) the proportion of the three sources with respect to total P uptake by ryegrass shoots during the two harvests. Each stack represents the mean values and error bars represent standard deviation. Different small letters refer to statistically significant differences, within each P source, while different capital letters represent statistically significant differences in total P uptake (Tukey HSD, p < 0.05). The arrows above the columns representing biochars indicate a statistically significant linear relationship with pyrolysis temperature.

Total P uptake ranged from 7.8 to 25.6 mg kg^−1^ soil (Figure 3A). Uptake was lowest in NoP (8.5 ± 0.5 mg kg^−1^ soil) and highest in MinP (24.8 ± 0.8 mg kg^−1^ soil). Among BDF‐derived biochars, BB400 had the highest uptake, with a significant decline at higher temperatures (BB600 < BB400; *p* < 0.05). In contrast, P uptake rates in PMF‐derived biochars were statistically similar (Tukey HSD, *p* > 0.05), with no significant relation between pyrolysis temperature and P uptake rates.

The calculated values of Pdf_seed_ were highest in NoP (3.6 ± 0.2 mg P kg^−1^ soil) and lowest in MinP (8.3 ± 0.2 mg P kg^−1^ soil) (Figure 3A), while Pdf_soil_ was similar among treatments, with a few exceptions. Statistically, Pdf_soil_ was significantly higher in PB600 than in NoP, PMF, BDF, BB500, and BB600 (Tukey HSD *p* < 0.05). The two control treatments with and without mineral fertilizer showed no significant differences in Pdf_soil_.

The treatments showed considerable differences in Pdf_fertilizer_ (Figure 3A). The highest Pdf_fertilizer_ was recorded in MinP, followed by BB400, BDF and PB400, BB500, PMF and PB500, and BB600 and PB600. The biochars produced at 400°C had significantly higher Pdf_fertilizer_ than the biochars produced at 600°C. Overall, there was a gradual decrease in average Pdf_fertilizer_ among biochars produced from the same feedstock as pyrolysis temperature increased.

Accordingly, the proportions of the three sources of P differed among the treatments (Figure 3B). The lowest and highest proportions of Pdf_seed_ and Pdf_soil_ were recorded in the two control treatments with and without mineral P addition to the soils, respectively. The proportion of Pdf_seed_ was similar in the pots amended with the solid fractions of biogas digestate and pig manure and their biochars. The highest proportion of Pdf_soil_ in organic fertilizers was found in PB600 followed by PB500. On the other hand, the proportion of Pdf_fertilizer_ in relation to P uptake was the highest in MinF, followed by BB400. The proportion of Pdf_fertilizer_ was significantly lower in the treatments amended with PMF and its biochars than MinP. In contrast, PB600 had the lowest proportion of Pdf_fertilizer_ relative to total P uptake compared to all treatments except PB500 and BB600.

### 3.4 FPR rates and P‐FRV

**FIGURE 4:**
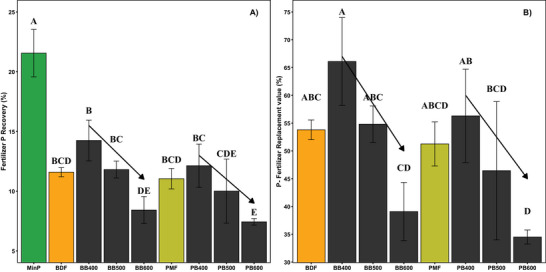
Fertilizer P recovery and P fertilizer replacement values for treatments with solid fractions of biogas digestate and pig manure or their biochars produced at different pyrolysis temperatures. Each bar represents the standard mean and error bars represent the standard deviation (*n* = 4). Bars with contrasting letters at the top are statistically different (Tukey HSD *p* < 0.05). The arrows above the columns representing biochars indicate a statistically significant linear relationship with pyrolysis temperature.

The highest FPR rates were evident in the MinP treatment, where more than 20% of the P supplied via MinP was taken up by the plant shoots (Figure 4A). Among the treatments receiving recycled P amendments, recovery rates were very similar in the treatments receiving the feedstocks and the biochars produced at 400°C and 500°C, respectively. The FPRs of the high‐temperature (600°C) biochars were the lowest of all treatments, ranging from 6% to 9%, and statistically different from those of the biochars produced at 400°C. No statistical differences were recorded when comparing the biochars from the two feedstocks (BDF and PMF) produced at the same pyrolysis temperature.

No statistically significant differences were found in the P‐FRVs of the BDF and PMF and their biochar produced at 400°C and 500°C (Figure 4B). The highest P‐FRV of 66 ± 8% was recorded in the biochar produced at 400°C. The mean P‐FRV of BDF and PMF was lower than that of the treatments with low‐temperature biochars (BB400 and PB400), but considerably higher than that of the treatments with high‐temperature biochars (BB600 and PB600). While a significant negative trend was evident between pyrolysis temperature and P‐FRV in both feedstock‐based biochars, the strength of this relationship varied considerably between feedstock types.

## 4 DISCUSSION

### 4.1 The quality of biochar as P fertilizer is affected by the pyrolysis temperature

Biochar as a soil amendment can influence soil P dynamics and plant uptake in several ways, including serving as a direct source of P. It can also enhance or reduce plant‐available P in the soil through its sorption capacity or by affecting microbial activities (Glaser & Lehr, 2019; Tesfaye et al., 2021). In the present study, we used an indirect ^33^P dilution approach to estimate the direct P uptake from biochars produced from the solid fractions of biogas digestate (BDF) and pig manure (PMF) at contrasting pyrolysis temperatures. Key parameters such as plant biomass (Figure 2), P uptake rates, Pdf_fertilizer_ (Figure 3), FPR, and P‐FRV (Figure 4) indicated that biochar could act as a P source just like the original P‐rich solid fractions (BDF and PMF). However, biochar produced at lower pyrolysis temperatures was more effective as a P fertilizer, as a negative trend was evident in the performance of biochar with increasing pyrolysis temperature (Figure 4).

The high potential of low‐temperature biochar as a P fertilizer has been suggested in the literature after assessing its effects on total P uptake by plants or by recording changes in plant‐available P extracted using methods such as DGT (diffusive gradients in thin films)‐P, Olsen‐P, and so forth (Gao et al., 2019; Glaser & Lehr, 2019; Tesfaye et al., 2021). For instance, Christel et al. (2014) recorded a significantly higher proportion of P measured by DGT in soils amended with low‐temperature biochar (up to 600°C) produced from the solid fraction of pig manure. Similarly, Bruun et al. (2017) also concluded that pyrolysis of the solid fraction of digestate at temperatures below 600°C had no effect on plant P availability despite changes in P speciation. The findings of the present study partially align with those of Christel et al. (2014) and Bruun et al. (2017). Like Bruun et al. (2017), we also observed a similar P uptake between the raw feedstock and biochar produced at low temperatures (Figure 3). However, we recorded a significantly lower P uptake in pots amended with biochar produced at 600°C (Figure 3). This discrepancy could be associated with the differences in the approach used between the studies, as the DGT‐based method may not fully predict plant uptake, or with differences in the composition of the original feedstocks that affected P speciation in the biochars.

The negative trend in performance availability in the biochar with increasing pyrolysis temperature is likely to be associated with changes in P speciation induced by the thermal treatment. We measured P contents in different fractions with variable solubility using sequential extractions to highlight changes in the proportions (Figure 1). Among the sequential fractions, WEP and bicarbonate‐P (combined), representing the P fractions readily available to plants (Cross & Schlesinger, 1995), showed a decreasing trend with increasing pyrolysis temperature (Figure 1). These findings align with those of W. Li et al. (2018), who evaluated biochar produced from poultry manure at pyrolysis temperatures ranging from 300°C to 600°C. Although the raw material used in W. Li et al. (2018) and the present study differed, both studies observed a significant decrease in WEP, an increase in HCl‐P, and an initial increase followed by a decrease in bicarbonate and NaOH fractions as pyrolysis temperature increased (Figure 1). That changes in P‐speciation were the primary factor behind the reduced performance of high‐temperature biochar was further supported by the significant positive linear correlation (*r* = 0.98, *p *= 0.0006) between the FPR and the combined proportion of WEP and bicarbonate‐P in the biochars used in this study (Figure S3). Similarly, the strong negative correlation between HCl‐P and P recovery (*r* = 0.94, p = 0.005; Figure S3) and the positive correlation between HCl‐P in biochar and pyrolysis temperature suggest the formation of complex P species that accumulate at higher temperatures. This aligns with Liu et al. (2023), who identified stable P crystals in manure‐derived biochar and their temperature‐dependent formation using X‐ray diffraction analysis.

A significant decrease in WEP following pyrolysis could have environmental benefits, as WEP is prone to leaching (Wang et al., 2016). Low‐temperature pyrolysis primarily converts WEP to bicarbonate P, which remains plant available but is less susceptible to leaching (Kleinman et al., 2002). However, low‐temperature biochars are often associated with lower carbon stability, and thus biochar from low‐temperature pyrolysis may represent a trade‐off between P availability and C sequestration (Woolf et al., 2021). Nevertheless, the low‐temperature biochar appears to be a valuable source of P for plant growth and has logistical advantages that allow longer recycling distances from animal farms and biogas production plants due to reduced transport costs.

As the present study shares the inherent limitations of pot experiments, including short‐term evaluation, uniformity in nutrients mixing, exaggerated plant/root density, comparatively homogeneous distribution of the fertilizer in the soil, and unaccounted residual P dynamics, we recommend further long‐term field evaluation of P‐rich biochar, along with assessments of economic and logistic feasibility of the separation and pyrolysis approach.

### 4.2 Feedstock‐dependent temperature responses in biochar P availability: BDF versus PMF

Despite significant differences in readily available P fractions (WEP and bicarbonate‐P), the two feedstocks (BDF and PMF) had comparable mean P‐FRV (Figures 1 and 4). The high abundance of residual P in BDF likely reflects its greater organic P content, which was not quantified by the inorganic‐P extraction approach, and this organic fraction probably supported early plant P uptake, as temporary N immobilization was observed in both treatments, which was likely a consequence of rapid organic matter mineralization. Because sequential extractions were performed on dried material, and drying can shift P speciation (Pantelopoulos et al., 2017), these raw material values may not truly represent the wet feedstocks applied in the pots.

Biochars produced from BDF and PMF at equivalent pyrolysis temperatures likewise showed overall similar P‐FRV values despite significant differences in the proportions of WEP and bicarbonate‐P (Figures 1 and 4), suggesting that anaerobic digestion of manure does not substantially affect the plant P availability of the resulting biochars. Feedstock effects on P‐FRV typically become more apparent when broader categories of biomass are compared (e.g., wood vs. cereal crops, straw vs. animal manures) (Glaser and Lehr, 2019; Tesfaye et al., 2021).

However, linear regression analyses revealed stronger temperature dependencies for BDF‐derived biochars than for those derived from PMF across most measured variables. This was mainly driven by the strongly reduced P availability in BB600 and also reflected in a significantly increased residual P fraction compared to PB600 in the sequential extraction (Figure 1). These differences likely arise from distinct elemental compositions of the raw feedstocks. W. Li et al. (2018) also observed an increased insoluble residual P fraction in poultry manure‐derived biochar at higher pyrolysis temperatures. They attributed this to the presence of available Ca, which was also much higher in BDF than in PMF. This highlights the complex interplay between biomass composition, thermal decomposition chemistry, and resulting biochar characteristics (Bai et al., 2023; Yu et al., 2024).

### 4.3 Indirect role of biochar on soil P availability and limitations of the ^33^P dilution approach

In addition to serving as a direct source of plant‐available P, biochar also plays an indirect role by influencing soil P dynamics (F. Li et al., 2019; Yang et al., 2021). However, due to methodological limitations, these two roles are difficult to separate. Consequently, most studies assess the effects of biochar application by measuring differences in total P uptake by plants or changes in soil P fractions known to be readily available to plants, for example, DGT‐P, Olsen‐P, and so forth, relative to a non‐amended control (Glaser and Lehr, 2019; Tesfaye et al., 2021). In this study, we attempted to untangle some of the direct and indirect effects by using the ^33^P dilution approach, which is based on labeling the available P pool present in the soil prior to the addition of an external P source. However, potential effects of biochar application on less available, non‐labeled inorganic or organic soil P would also dilute the specific activity in plants from the biochar‐amended treatments and overestimate the contribution from the fertilizer. To minimize this effect during the experiment, the incubation of the soil for 1–2 weeks after addition of the ^33^P solution is recommended (Oberson et al., 2010) and was adopted in this study. Moreover, we reduced the soil P content, including organic P, by using a soil low in total P and organic matter and adding 25% of sand. However, the potential release of P from any of the abovementioned potential sources, particularly driven by the changes after biochar application, cannot be completely ignored and should thus be considered as a limitation of the study.

There were no significant differences in Pdf_soil_ between all biochar types and the negative control except for PB600 (Figure 3A), suggesting that most biochars either did not interact with soil P to a noticeable extent or had a similar interaction ability. One crucial factor influencing P availability, which is also affected by biochar amendment, is soil pH (Yang et al., 2021). However, since all biochar treatments exhibited similar liming effects (Figure S4), the elevated Pdf_soil_ in PB600 was unlikely to be driven by pH changes. Other biochar properties influencing direct interactions with soil P include surface area, porosity, surface functional groups, dissolved organic matter content, and the proportion of co‐precipitating ions such as Al, Fe, and Mg (F. Li et al., 2019). These factors, which affect P sorption, are primarily determined by pyrolysis temperature and feedstock type (Gui et al., 2020; Rodríguez Alberto et al., 2021). However, given the limited characterization data, we cannot pinpoint the specific property of PB600 responsible for its significantly higher Pdf_soil_. It is possible that PB600 had a lower P sorption potential than the other biochar types, leading to increased Pdf_soil_. A recent study reported a significantly lower phosphorus sorption capacity for farm manure‐based biochar produced at 600°C compared to that produced at 400°C (Musa et al., 2024).

An additional challenge with the ^33^P dilution approach is accounting for P derived from seed (Pdf_seed_), which is estimated by a mathematical function derived from a correlation between total P uptake and Pdf_seed_, using values from a parallel sand‐based experiment. In the case of perennial grass, for example, ryegrass, with multiple harvests to assess P uptake, the seed contribution is typically accounted for in the first two cuts (Meyer et al., 2017; Nanzer et al., 2014). However, in the present study we only considered the seed P contribution for the first cut, where it is most important, because at the time of the second cut, the seed P contribution for the high P treatment exceeded the amount of P added with the seeds in the sand experiment. This discrepancy may be attributed to the presence of residual P in the sand, a factor that is often overlooked (Brod, 2016). The calculations resulted in an estimated Pdf_seed_ in the NoP treatment that ranged from 90% to 98% of the P added via seeds in the first cut, while these estimates exceeded 100% in the remaining treatments. Therefore, the absolute amount of Pdf_seed_ is somewhat overestimated in our study. Similar issues have been observed in previous studies using the ^33^P dilution approach (Brod, 2016; V. Hansen et al., 2022; Meyer et al., 2017). The overestimation of Pdf_seed_ could result in an underestimation of the other P sources (Pdf_seed_ and Pdf_fertilizer_), which are calculated after subtracting Pdf_seed_. However, the similar trends in total P uptake and in Pdf_fertilizer_ between the treatments (Figure 2A) suggest that the potential overestimation of Pdf_seed_ did not affect the primary findings of this study. Nevertheless, the limitations of the ^33^P dilution approach should be acknowledged, as previously highlighted by Brod et al. (2016), and should be minimized by adopting alternative approaches proposed in the literature (Achat et al., 2014; Oberson et al., 2010).

## 5 CONCLUSION

Separation and pyrolysis of P‐rich biowaste, such as biogas digestate and pig manure, is a promising approach to reduce the environmental and health risks associated with their direct application to soil. The solid fraction of pig manure and biogas digestate showed a comparable P fertilizer effect in our study, which was about 50% of that of a mineral fertilizer. Pyrolysis decreased the proportion of water‐extractable P for the materials used in the present study, and this was accompanied by an increasing HCl‐extractable fraction with increasing temperature, indicating the formation of poorly soluble Ca‐phosphates. However, a similar P recovery and P‐FRV of low‐temperature biochars (400°C and 500°C) as their raw feedstocks suggests preservation of sufficient plant‐available P to support plant growth. Higher pyrolysis temperature reduced the P‐FRV, especially in the digestate‐derived biochar, predominantly due to a significant reduction in readily available P. Due to limitations of the ^33^P approach, we could not completely account for the effect of biochar on the release of native P from soil, although one high‐temperature biochar (PB600) significantly increased soil‐derived P uptake, which could be the subject of further investigations including diverse types of manures and biogas digestate. Given the inherent limitations of pot experiments, we recommend long‐term field trials to comprehensively evaluate the fertilizer value of nutrient‐rich biochars, including assessments of the additional plant nutrients (e.g., Ca, K) and their synergistic effects.

## AUTHOR CONTRIBUTIONS

**Saadatullah Malghani**: Data curation; formal analysis; investigation; writing—original draft. **Sander Bruun**: Conceptualization; funding acquisition; project administration; writing—review and editing. **Muhammad Ashfaq Wahid**: Investigation; writing—review and editing. **Dorette Sophie Müller‐Stöver**: Conceptualization; methodology; resources; supervision; validation; writing—original draft.

## CONFLICT OF INTEREST STATEMENT

The authors declare no conflicts of interest.

## Supporting information



Supplementary information
