# Supplementary material for: Impact of pyrolysis temperature on phosphorus plant availability in biochar—A pot experiment using 33P dilution
Source: J Environ Qual. 2025 Aug 30;54(6):1944–56. doi: 10.1002/jeq2.70075 (PMC12593314; doi:10.1002/jeq2.70075)
Supplement: Supplementary file 1 — Supplementary information [file JEQ2-54-1944-s001.docx]

***Supplementary Results***

**Impact of pyrolysis temperature on phosphorus plant availability in biochar – a pot experiment using ^33^P dilution**

Saadatullah Malghani^a^, Sander Bruun^a^, Muhammad Ashfaq Wahid^ab^, Dorette Sophie Müller-Stöver^a*^

^a^Department of Plant and Environmental Sciences, Faculty of Science, University of Copenhagen, Denmark

^b^ Department of Agronomy, University of Agricultural Faisalabad, Pakistan

*Corresponding author: dsst@plen.ku.dk

**Table S1.** Molar ratio of key elements with respect to total P in solid fractions of biogas digestate (BDF), pig manure (PMF) and their biochars produced at contrasting pyrolysis temperatures.

| **Material** | **Ca:P** | **Mg:P** | **Al:P** | **Fe:P** |
| --- | --- | --- | --- | --- |
| **BDF** | 2.60 ± 0.2^b^ | 1.22 ± 0.02^b^ | 0.13 ± 0.01^a^ | 0.16 ± 0.01^a^ |
| **BB400** | 2.89 ± 0.02^ab^ | 1.27 ± 0.01^a^ | 0.14± 0.01^a^ | 0.17 ± 0.01^a^ |
| **BB500** | 2.89 ± 0.02^a^ | 1.28 ± 0.02^a^ | 0.14± 0.01^a^ | 0.17 ± 0.01^a^ |
| **BB600** | 2.89 ± 0.01^a^ | 1.28 ± 0.02^a^ | 0.13 ± 0.01^a^ | 0.17 ± 0.01^a^ |
| **PMF** | 1.8 ± 0.1^c^ | 0.85 ± 0.02^c^ | 0.06 ± 0.01 ^b^ | 0.09 ± 0.01^b^ |
| **PB400** | 1.9 ± 0.01^c^ | 0.86 ± 0.01^c^ | 0.06 ± 0.01 ^b^ | 0.10 ± 0.01^b^ |
| **PB500** | 2.0 ± 0.01^c^ | 0.86± 0.01^c^ | 0.05 ± 0.01^b^ | 0.10 ± 0.01^b^ |
| **PB600** | 2.0 ± 1.03^c^ | 0.88 ± 0.01^c^ | 0.05 ± 0.01^b^ | 0.10 ± 0.01^b^ |

Values represent mean±sd n = 4 except PB600 (3 replicates)

**Table S2** Linear regression analysis of pyrolysis temperatures (400, 500, 600°C) with elemental concentrations in biochars derived from solid fractions of digestate (BDF) and pig manure (PMF)

| Parameter | Digestate solids biochar | | | Pig manure solids biochar | | |
| --- | --- | --- | --- | --- | --- | --- |
|  | R^2^ | *p-*value | Trend direction | R^2^ | *p*-value | Trend direction |
| TC | 0.96 | <0.001 | ↑ | 0.85 | <0.01 | ↑ |
| TN | 0.901 | <0.01 | ↓ | 0.81 | <0.05 | ↓ |
| Ash | 0.91 | <0.01 | ↑ | 0.92 | <0.01 | ↑ |
| Ca contents | 0.82 | <0.01 | ↑ | 0.42 | ns | - |
| Mg contents | 0.81 | <0.01 | ↑ | 0.47 | ns | - |
| Al contents* | 0.798 | <0.01 | ↑ | 0.38 | ns | - |
| Fe contents* | 0.42 | ns | - | 0.39 | ns | - |
| TP contents* | 0.82 | <0.01 | ↑ | 0.38 | ns | - |
| WEP | 0.95 | <0.001 | ↓ | 0.81 | <0.001 | ↓ |
| Biocarbonate-P | 0.56 | <0.05 | ↓ | 0.26 | ns | - |
| NaOH-P | 0.88 | <0.001 | ↓ | 0.89 | <0.001 | ↓ |
| HCl-P | 0.97 | <0.001 | ↑ | 0.93 | <0.001 | ↑ |

*Values were log transformed


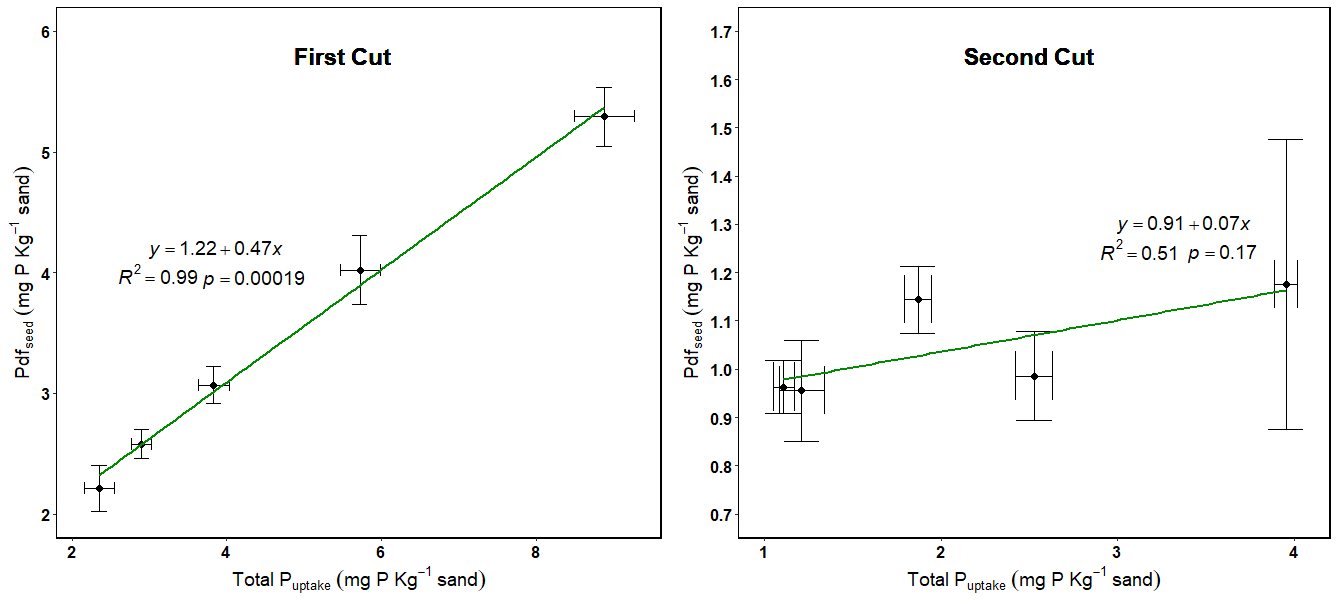


**Figure S1**. Relation between the shoot P uptake and the seed P contribution (Pdf _seed_) for the

two cuts of Italian ryegrass grown on sand and fertilized with different rates of water soluble

P labeled with ^33^P. The relation was used to estimate the P derived from the seed (Pdf_seed_)

in plants grown on soil. Error bars present standard deviations of replicates (n=4)


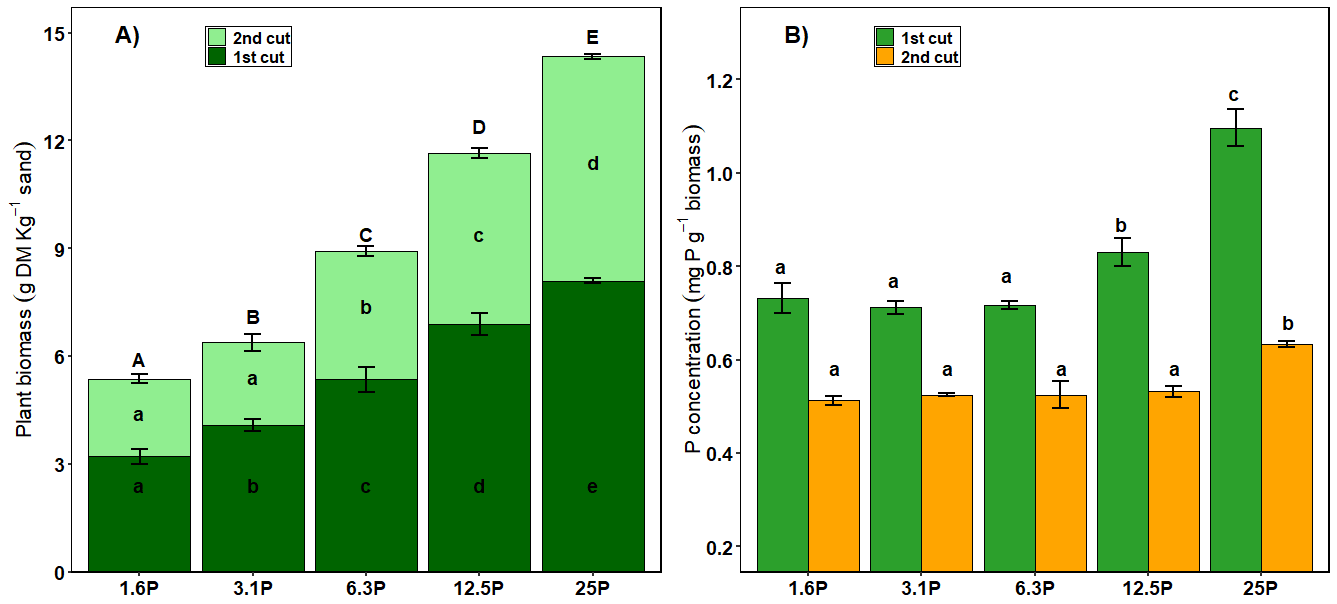


**Figure S2.** Plant biomass and its P concentrations among sand experiment to record P derived from seed. (A) Dry matter contents of Ryegrass grass harvested twice. Each stack represents the mean value of each harvest and error bars represent standard deviation. (B) P concentration of Ryegrass biomass at each harvest. Different letters at the top of each graph or within each stack represent statistical significance among treatments (Tukey HSD Test, α=0.05).


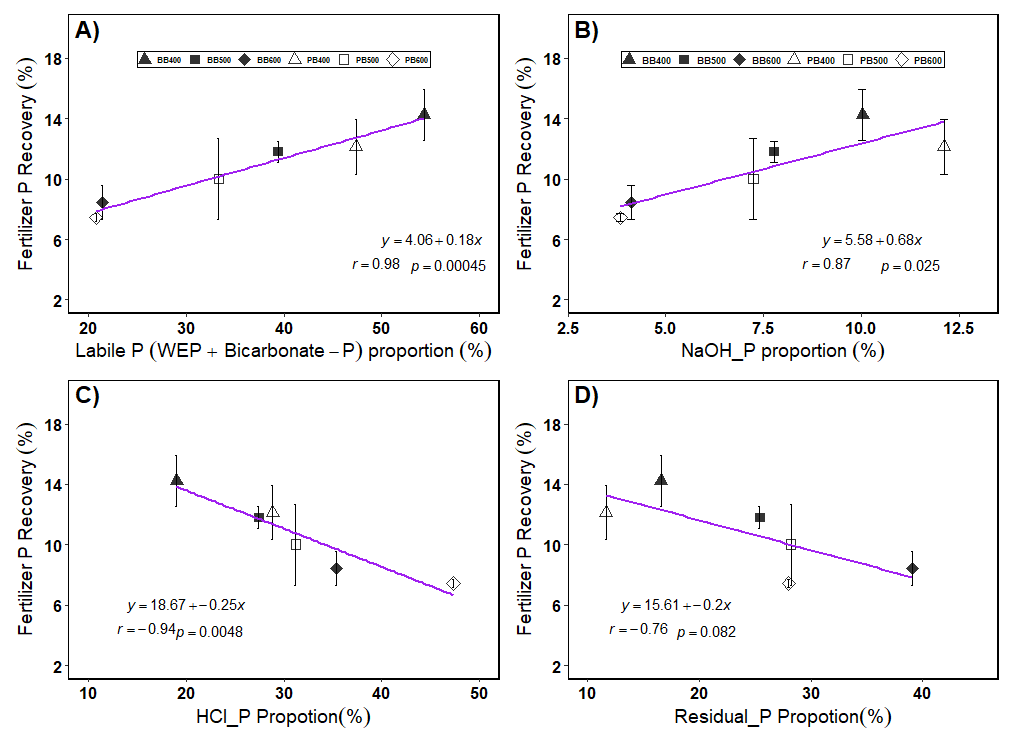


**Figure S3.** Linear correlation of Fertilizer P Recovery with the proportion of different Headley sequential P fractions in biochar produced from solid fractions of biogas digestate and pig manure at 400, 500 or 600°C pyrolysis temperature.


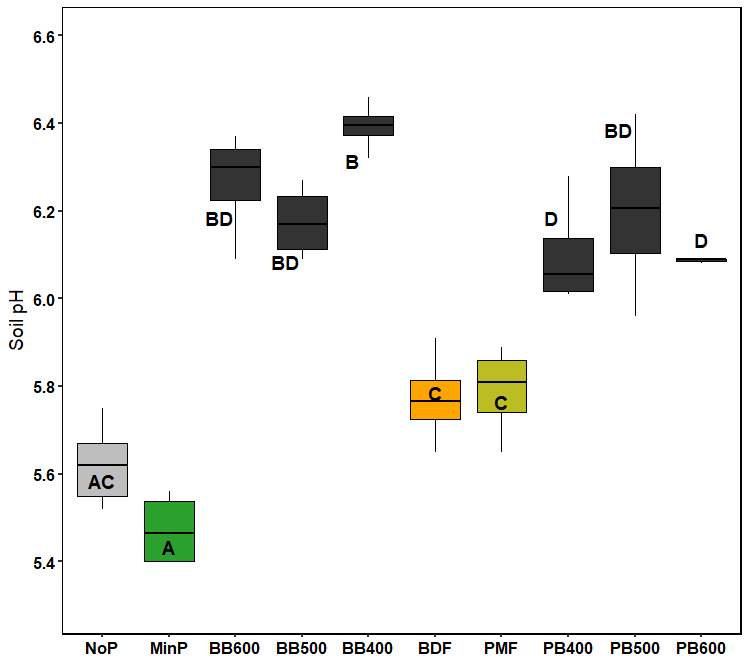


**Figure S4.** Box plot representing Soil pH measured at the end of the pot experiment (1:5 soil water ratio). Boxed with contrasting alphabet are statistically significantly different among each other (HSD tukey α=0.05)
